# Hospitals’ uneven recovery from the COVID-19 pandemic

**DOI:** 10.1093/haschl/qxad034

**Published:** 2023-08-17

**Authors:** Jordan H Rhodes, Tatiane Santos, Gary J Young

**Affiliations:** Maine Department of Health and Human Services, Augusta, ME 04333, United States; Tulane University School of Public Health and Tropical Medicine, New Orleans, LA 70112, United States; Center for Health Policy and Healthcare Research, Northeastern University, Boston, MA 02115, United States; D’Amore-McKim School of Business, Northeastern University, Boston, MA 02115, United States; Bouve College of Health Sciences, Northeastern University, Boston, MA 02115, United States

**Keywords:** health economics, hospital finances, COVID-19

## Abstract

Using hospital cost report data from the Centers for Medicare and Medicaid Services, we examined the changes in hospitals’ operating margins and total margins between 2019 and 2021. We found that, as of 2021, hospitals’ operating margins had not fully rebounded to 2019 levels, although they had recovered from the 2020 nadirs. Conversely, average total margins increased by 140% during this period across all US hospitals, with the most significant growth occurring among rural hospitals, publicly operated hospitals, and critical access hospitals. Rural hospitals exhibited the largest gains in total margins during this time, experiencing a 1600% increase from 2019 to 2021. Our findings indicate that government relief funding tied to the COVID-19 Public Health Emergency significantly bolstered the financial health of the average hospital and had an outsize effect on lifting total margins among smaller hospitals that entered the pandemic in the most financially vulnerable position.

## Introduction

The onset of the COVID-19 pandemic had a devastating impact on hospitals’ operating margins, the result of substantial declines in revenues linked to patient care and a surge in operating expenses.^1,2^ Despite this, many hospitals are emerging from the pandemic in a more financially stable position than they entered.^3^ An important policy question arises from this seemingly contradictory finding: which hospitals exhibited the largest financial gains during the pandemic and why? To answer this question, we used publicly available data from the Centers for Medicare and Medicaid Services (CMS) to examine changes in key financial indicators over time for all US hospitals, as well as for a subset of financially vulnerable hospitals: rural hospitals, public hospitals, and critical access hospitals.

Our study is most closely related to 2 recent studies that examined the impact of the pandemic on hospitals’ financial outcomes through 2020.^1,2^ Our analysis diverges from these studies in several ways. First, we used more recent data from CMS to examine hospitals’ 2021 financial outcomes. We found that, as of 2021, hospitals’ operating margins had not fully rebounded to 2019 levels, although they had recovered from the 2020 nadirs. Conversely, average total margins increased by 140% across all US hospitals during this period.

Second, we extended analysis and findings from prior studies to identify the primary factors underlying the heterogenous growth in total margins across hospitals since the onset of the pandemic.^1,2^ We document a significant increase in average levels of non–operating income between 2019 and 2021. While the average increase in non–operating income corresponding to financially vulnerable hospitals is lower in nominal terms relative to the average hospital, this additional revenue has an outsize effect on lifting 2021 total margins among these hospitals. This effect is most pronounced among rural hospitals and public hospitals, given the small scale at which these facilities operate. As a result, the growth in average total margins for financially vulnerable hospitals far outpaced the national average, approaching 400% between 2019 and 2021. Rural hospitals saw the largest gains in total margins during this time, experiencing a 1600% increase from 2019 to 2021.

Third, using a newly available field in the CMS data, the amount of COVID-19 Public Health Emergency (PHE) funding received, we further examined the growth in non–operating income during this period by calculating what 2020 and 2021 total margins would have been had hospitals not received government funding tied to the COVID-19 PHE. We found that, even in the absence of this funding, hospitals’ 2021 total margins would have exceeded pre-pandemic levels, on average. Our findings suggest that government relief funding tied to the PHE significantly bolstered the financial health of the average hospital in 2021. The outsize effect that this funding had on rural hospitals may have contributed to the deceleration of rural hospital closures in recent years.^4^

## Data and methods

The primary source of data for our analysis derives from publicly available hospital cost report data from CMS for the period of 2017 to 2021.^5^ All hospitals that receive payments from the Medicare program are required to submit cost report data on an annual basis in the form of standardized worksheets. Our analysis comprised all general acute care and critical access hospitals. The cost report data have several well-documented limitations, including missing and inaccurate submissions. We used rigorous data-cleaning practices that have been developed and implemented in prior studies to address data inconsistencies.^2,6,7^ Our data-cleaning methods included aggregating financial outcomes in instances where a hospital submitted multiple reports corresponding to nonoverlapping periods within the same year; flagging, examining, and removing infeasible outlier observations; and, scrutinizing geographic indicators, including hospital state, county, and zip code fields. We converted all financial outcomes into 2021 dollars to account for inflation and to facilitate comparability across data years. We relied on hospital ownership information provided in the cost report data to identify publicly operated facilities. We linked to the cost report data the following county-level data: rural–urban continuum codes from the US Department of Agriculture, uninsured rates and poverty rates from the Census Bureau, and unemployment rates from the Bureau of Labor Statistics.^8–11^

A key difference between operating margins and total margins is the inclusion of non–operating income in the calculation of total margins. This includes various sources of revenues and expenses not directly tied to patient care, such as contributions, investments, and government appropriations. Beginning in 2020, funding linked to the COVID-19 PHE, including the Provider Relief Fund, was incorporated into this calculation.

Table 1 provides baseline information pertaining to institutional, county, and financial outcomes for the full sample of hospital cost report submissions with a fiscal year that ended in 2019 (n = 4221). Because hospitals report on a fiscal year basis, and most hospitals do not have a fiscal year that aligns with the calendar year, our analysis is conducted based on the calendar year that corresponds to a hospital's fiscal year end date. For example, a hospital cost report submission with a fiscal year spanning October 2018 through September 2019 is assigned to data year 2019, whereas a hospital with a fiscal year spanning April 2019 through March 2020 is assigned to data year 2020. Across all hospitals in our sample, the average reporting period end month is 9, indicating that, on average, hospitals have a fiscal year spanning September through August.

**Table 1.** Baseline (2019) hospital institutional, county, and financial characteristics.

A. 2019 sample and institutional characteristics						
	No.	Mean reporting period end month	Financially vulnerable hospitals	% Rural	% Public	% Critical access
All hospitals	4221	8.8	40.5%	7.9%	22.0%	30.4%
Financially vulnerable hospitals	1708	8.4	100%	19.5%	54.4%	75.1%
Rural hospitals	333	8.3	100%	100%	48.4%	92.2%
Public hospitals	929	8.3	100%	17.3%	100%	55.8%
Critical access hospitals	1283	8.6	100%	23.9%	40.4%	100%

B. 2019 mean hospital county characteristics			
	Uninsured rate	Unemployment rate	Poverty rate
All hospitals	11.2%	3.8%	13.4%
Financially vulnerable hospitals	11.9%	3.9%	14.2%
Rural hospitals	12.9%	3.8%	14.4%
Public hospitals	12.8%	3.9%	14.6%
Critical access hospitals	11.6%	3.9%	13.7%

C. 2019 mean hospital financial outcomes						
	Net patient revenues ($ millions)	Operating expenses ($ millions)	Other income ($ millions)	Other expenses ($ millions)	Operating margins	Total margins
All hospitals	$238.85	$240.00	$21.70	$1.58	−0.0445	0.0413
Financially vulnerable hospitals	$109.95	$119.52	$16.13	$0.98	−0.1124	0.0224
Rural hospitals	$21.18	$22.23	$2.11	$0.10	−0.1353	0.0080
Public hospitals	$172.38	$189.55	$27.47	$1.50	−0.1661	0.0200
Critical access hospitals	$31.09	$32.32	$3.10	$0.45	−0.0945	0.0223

Source: 2016–2021 Healthcare Cost Report Information System Cost Report Data; US Department of Agriculture; US Census Bureau; US Bureau of Labor Statistics.^5,8–11^ All financial outcomes appear in 2021 dollars.

In addition to reporting summary statistics for the full sample of US hospitals, Table 1 also includes summary statistics corresponding to the subset of financially vulnerable hospitals. Financially vulnerable hospitals constitute approximately 41% of all hospitals in our sample. Relative to all US hospitals, financially vulnerable hospitals are in counties with higher average uninsured rates, unemployment rates, and poverty rates. Prior to the onset of the pandemic, financially vulnerable hospitals operated at a much smaller scale than the average US hospital, with 2019 operating expenses of approximately half the national average ($120 million vs $240 million). On average, non–operating income constituted a greater percentage of total revenues for financially vulnerable hospitals (13%) relative to the full sample of hospitals (8%). Significant heterogeneity existed within categories of financially vulnerable hospitals for both types of margins. While public hospitals reported the lowest average operating margins in 2019 (−17%), rural hospitals had the lowest total margins at approximately 1%.

To assess how hospitals are emerging from the pandemic financially, we examined changes in average margins outcomes for the period 2017 through 2021. We then focused our analysis on the changes in margins outcomes between 2019 and 2021: 1 year prior to the onset of the pandemic and 1 year following the most acute effects of the pandemic. We conducted 2-sided *t* tests to compare the average components of margins outcomes across years. We clustered standard errors at the state-level to allow for correlation across hospitals within the same state over time.

## Results

### Changes in margins outcomes

**Figure 1.**
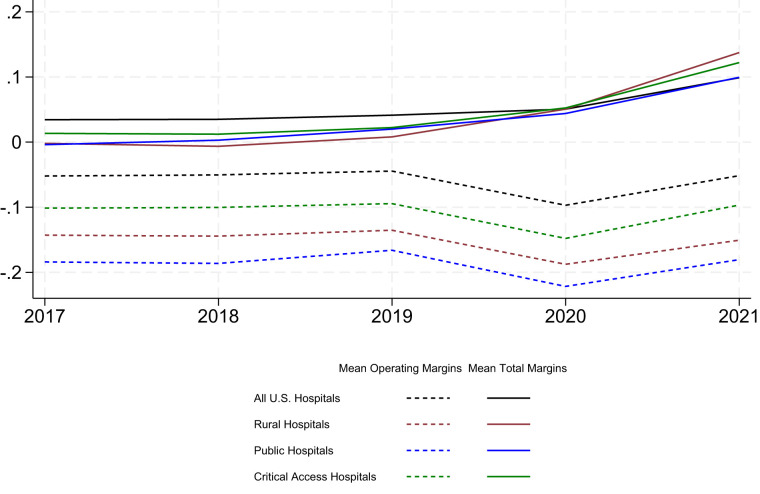
Changes in operating margins and total margins over time (2017–2021) by hospital institutional characteristics. Source: 2016-2021 Healthcare Cost Report Information System Cost Report Data; US Department of Agriculture.

Figure 1 presents the changes in average margins over time for each of the 3 categories of financially vulnerable hospitals, as well as the full sample of US hospitals. Across all 3 categories of financially vulnerable hospitals and the full sample of hospitals, average operating margins declined substantially between 2019 and 2020, while average total margins increased. The growth in total margins from 2019 to 2020 is most pronounced among rural hospitals, followed by critical access hospitals and public hospitals. Across all categories of hospitals, 2021 average operating margins rebounded from 2020 low points, while 2021 average total margins continued to grow from the 2020 levels.

We examined the sensitivity of these findings to varying hospital fiscal year reporting periods. We stratified our analysis by hospitals with fiscal year reporting periods that begin in either January, July, or October; these hospitals constitute more than 90% of all hospitals in our sample. The results from this analysis are presented in Appendix Exhibit S1. We found that the changes in margins over time observed across these subsamples of hospitals largely resemble the changes observed across all hospitals. Relative to hospitals with a fiscal year beginning in July or October, hospitals with a fiscal year beginning in January experienced a greater increase in total margins beginning in 2020, likely driven by the fact that their fiscal year more closely overlaps with the time frame for the distribution of funding. This finding aligns with Wang et al,^1^ who focused their analysis on the subset of hospitals with a fiscal year that spans the calendar year; they documented comparable increases in total margins from 2019 to 2020.

### Changes in financial components of margins

We examined the components of operating margins and total margins to determine the factors underlying the changes in these financial indicators from 2019 to 2021. Table 2 presents the changes in net patient revenues, operating expenses, other income, other expenses, as well outcomes for both margins, during this period. While only net patient revenues and operating expenses factor into the calculation of operating margins, all 4 variables are used in the calculation of total margins. Across the full sample of US hospitals, as well as all 3 categories of financially vulnerable hospitals, both net patient revenues and operating expenses increased from 2019 to 2021. The growth in operating expenses during this period may have been partially driven by increased administrative expenses incurred during the pandemic.^12^ The growth in expenses generally exceeds the growth in revenues from 2019 to 2021; this aligns with the finding that, while 2021 operating margins rebounded from the 2020 low points, they remained below pre-pandemic levels.

**Table 2.** Changes in mean financial outcomes from 2019 to 2021.

	Net patient revenues ($ millions)	Operating expenses ($ millions)	Other income ($ millions)	Other expenses ($ millions)	Operating margins	Total margins
All US hospitals						
2019 Average	$238.85	$240.00	$21.70	$1.58	−0.0445	0.0413
2021 Average	$244.05	$248.20	$34.95	$0.72	−0.0516	0.0989
Change (2019 to 2021)	$5.19	$8.19	$13.25	−$0.87	−0.0071	0.0576
*P* value	.25	.08	<.01	<.01	.12	<.01
Percent change (2019 to 2021)	2.17%	3.41%	61.06%	−54.63%	−15.85%	139.34%
Financially vulnerable hospitals						
2019 Average	$109.95	$119.52	$16.13	$0.98	−0.1124	0.0224
2021 Average	$111.08	$122.82	$25.34	$1.04	−0.1170	0.1104
Change (2019 to 2021)	$1.13	$3.29	$9.21	$0.05	−0.0046	0.0880
*P* value	.66	.21	<.01	.83	.46	<.01
Percent change (2019 to 2021)	1.03%	2.76%	57.14%	5.37%	−4.13%	393.21%
Rural hospitals						
2019 Average	$21.18	$22.23	$2.11	$0.10	−0.1353	0.0080
2021 Average	$22.02	$23.44	$6.20	$0.06	−0.1506	0.1374
Change (2019 to 2021)	$0.85	$1.21	$4.09	−$0.04	−0.0153	0.1295
*P* value	<.01	<.01	.01	.16	.09	<.01
Percent change (2019 to 2021)	4.00%	5.46%	193.33%	−36.13%	−11.31%	1624.21%
Public hospitals						
2019 Average	$172.38	$189.55	$27.47	$1.50	−0.1661	0.0200
2021 Average	$174.72	$196.22	$42.14	$1.71	−0.1805	0.0995
Change (2019 to 2021)	$2.34	$6.67	$14.67	$0.20	−0.0144	0.0795
*P* value	.61	.16	<.01	.66	.10	<.01
Percent change (2019 to 2021)	1.36%	3.52%	53.39%	13.58%	−8.70%	398.24%
Critical access hospitals						
2019 Average	$31.09	$32.32	$3.10	$0.45	−0.0945	0.0223
2021 Average	$32.69	$33.93	$6.42	$0.43	−0.0966	0.1221
Change (2019 to 2021)	$1.60	$1.61	$3.32	−$0.02	−0.0022	0.0998
*P* value	.02	<.01	<.01	.82	.76	<.01
* * Percent change (2019 to 2021)	5.16%	4.98%	107.08%	−3.56%	−2.30%	448.14%

Source: 2016–2021 Healthcare Cost Report Information System Cost Report Data; US Department of Agriculture. All financial outcomes appear in 2021 dollars.

The primary driver underlying the growth in total margins from 2019 to 2021 is nonoperating sources of income. Across the full sample of hospitals, non–operating income grew from an average of $22 million in 2019 to $35 million in 2021; this constitutes a 62% increase during this period. Across rural hospitals, non–operating income increased by an average of $4 million. While this increase is significantly below the average increase observed across all US hospitals in nominal terms, it represents a 193% increase from the baseline mean of $2 million for rural hospitals. Given the small scale at which these hospitals operate, this funding had an outsized impact on lifting total margins. Rural hospitals’ nonpatient expenses also declined, on average, by 36% from 2019 to 2021, but this equates to an average reduction of only $40 000.

### The impact of PHE funding on total margins

**Figure 2.**
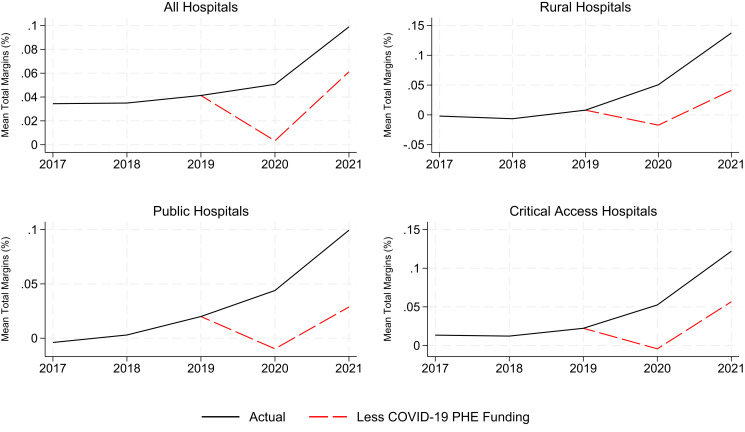
Total margins over time (2017–2021) including and excluding Public Health Emergency (PHE) funding by hospital institutional characteristics. Source: 2016-2021 Healthcare Cost Report Information System Cost Report Data; US Department of Agriculture.

We further examined the primary driver underlying the changes in total margins from 2019 to 2021 by assessing the impact of government funding tied to the COVID-19 PHE, a component of non–operating income. The results from this analysis are presented in Figure 2, which contains average total margins (inclusive of PHE funding) and average total margins that exclude this funding. We found that hospitals’ average total margins declined substantially between 2019 and 2020 when excluding funding tied to the PHE, indicating that these funds kept hospitals financially afloat following the onset of the pandemic. However, by 2021, hospitals’ average total margins had recovered, and even exceeded pre-pandemic levels, when excluding funding tied to the PHE. This finding is consistent across all 3 categories of financially vulnerable hospitals. The substantial gap in 2021 margins outcomes observed among rural hospitals, public hospitals, and critical access hospitals highlights the outsize effect that this funding had on lifting total margins among these hospitals.

As a sensitivity analysis, we also calculated total margins that exclude income from investments to discern whether the growth in market performance in recent years may have contributed to the increase in total margins during this period. The results from this analysis are presented in Appendix Exhibit S2. We found that income earned through investments does not explain the growth in total margins from 2019 to 2021, as evidenced by the relatively small gap between margin outcomes during this period. Relative to the average hospital, the gap between these outcomes is less pronounced among rural, public, and critical access hospitals, indicating that income from investments had a smaller effect on these hospitals’ financial health during the period of analysis.

## Discussion

The findings from our study indicate that, on average, hospitals’ overall financial health improved during the first 2 years of the pandemic, with substantial growth in the second year. This appears to be the direct result of government funding linked to the PHE. Hospitals that were most likely to enter the pandemic in a financially precarious position—rural, public, and critical access hospitals—experienced the largest growth in total margins during this time. The financial lift stemming from government relief funding over the past 2 years may serve to preserve and sustain access to health care in the areas in which these hospitals operate. This bolstered financial position has likely contributed to the deceleration of rural hospital closures in recent years.^4^

Our results offer a more nuanced view to the debate over the distribution of PHE funding to hospitals. Recent reporting suggests that the distribution of provider relief funds, which were intended to sustain hospitals through the pandemic, was ill targeted.^13,14^ While the findings from our analysis confirm that this funding bolstered, rather than merely sustained, total margins across many hospitals, the distribution of funding does seem to have significantly benefitted those hospitals that entered the pandemic in the most financially vulnerable position.

Notably, rural hospitals experienced the largest gains in total margins from 2019 to 2021. According to a recent Medicaid and CHIP Payment Access Commission (MACPAC) report, the Department of Health and Human Services extended additional provider relief funds to address the shortcomings of the initial general distribution.^15^ While the initial distribution of funding was solely based on hospitals’ net patient revenue, subsequent funding mostly targeted rural providers, safety-net hospitals, and nursing homes. Our findings align with the report's conclusion that rural hospitals and critical access hospitals received more relief funds as a share of their operating expenses relative to other hospitals. Even within categories of financially vulnerable hospitals, we found that hospitals with the lowest total margins in 2019 tended to experience the most significant gains from 2019 to 2021 (Appendix Exhibit S3). This relationship persists even after controlling for a robust set of hospital and geographic characteristics, including fiscal year start month; indicators for rural hospital, public hospital, and critical access hospital classification; hospital region indicators; hospital state indicators; and exposure to the pandemic (Appendix Exhibit S4).^16^ Across all models, both the magnitude and the precision of the estimate corresponding to baseline (2019) total margins are largely unchanged.

As an additional consideration, we note that while, on average, hospitals’ overall financial health improved between 2019 and 2021, many facilities may be facing significant financial obstacles in the near future as government funding linked to the pandemic comes to an end. Early reports suggest that hospitals continued to experience high labor costs throughout 2022 and early 2023.^17^ Furthermore, millions of individuals stand to lose Medicaid coverage with the unwinding of the continuous enrollment provision that was established during the pandemic. A recent report by the Congressional Budget Office estimated that approximately 15.5 million individuals will lose their Medicaid coverage over the next 18 months and that 6.2 million of these individuals will end up uninsured.^18^ The imminent increase in uninsurance rates will likely translate into increased levels of hospital uncompensated care costs and lower operating margins.^19^ These effects may be felt most significantly in communities served by rural, public, and critical access hospitals. Despite these challenges, hospitals that entered the pandemic in a financially precarious state may be able to shape their future financial health depending on how they invest and use the relief funding that they received. This will be an important topic for future research.

## Conclusion

The results from this study indicate that government funding tied to the COVID-19 PHE significantly bolstered many hospitals’ financial position. Notably, hospitals that tended to enter the pandemic in poor financial health—rural hospitals, public hospitals, and critical access hospitals—exhibited the largest gains in total margins from 2019 to 2021, despite maintaining relatively low operating margins. Funding distributed during the PHE may serve to slow or halt the pace of hospital closures and consolidation in areas where these facilities operate; however, this will depend on how hospitals invest and use these funds, as well as external market factors.

## Supplementary Material

qxad034_Supplementary_Data
