# Supplementary material for: Hospitals’ uneven recovery from the COVID-19 pandemic
Source: Health Aff Sch. 2023 Aug 17;1(3):qxad034. doi: 10.1093/haschl/qxad034 (PMC10986231; doi:10.1093/haschl/qxad034)
Supplement: qxad034_Supplementary_Data [file qxad034_Supplementary_Data.zip › APPENDIX 9.11.2023.pdf]

EXHIBIT S1: Changes in Margins Over Time (2017-2021) by Fiscal Year Start Month

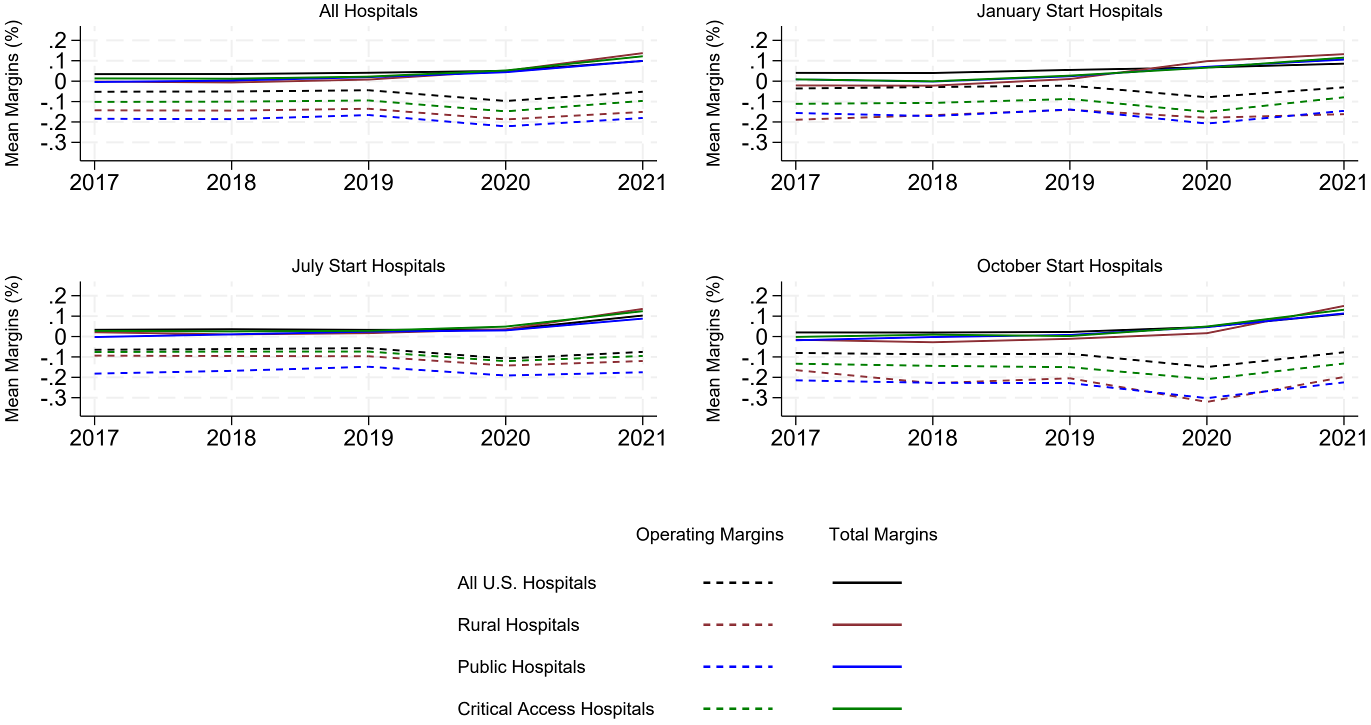

Sources: 2016-2021 HCRIS Cost Report Data; U.S. Department of Agriculture.

EXHIBIT S2: Total Margins Over Time (2017-2021) Including and Excluding Income from Investments
by Hospital Institutional Characteristics

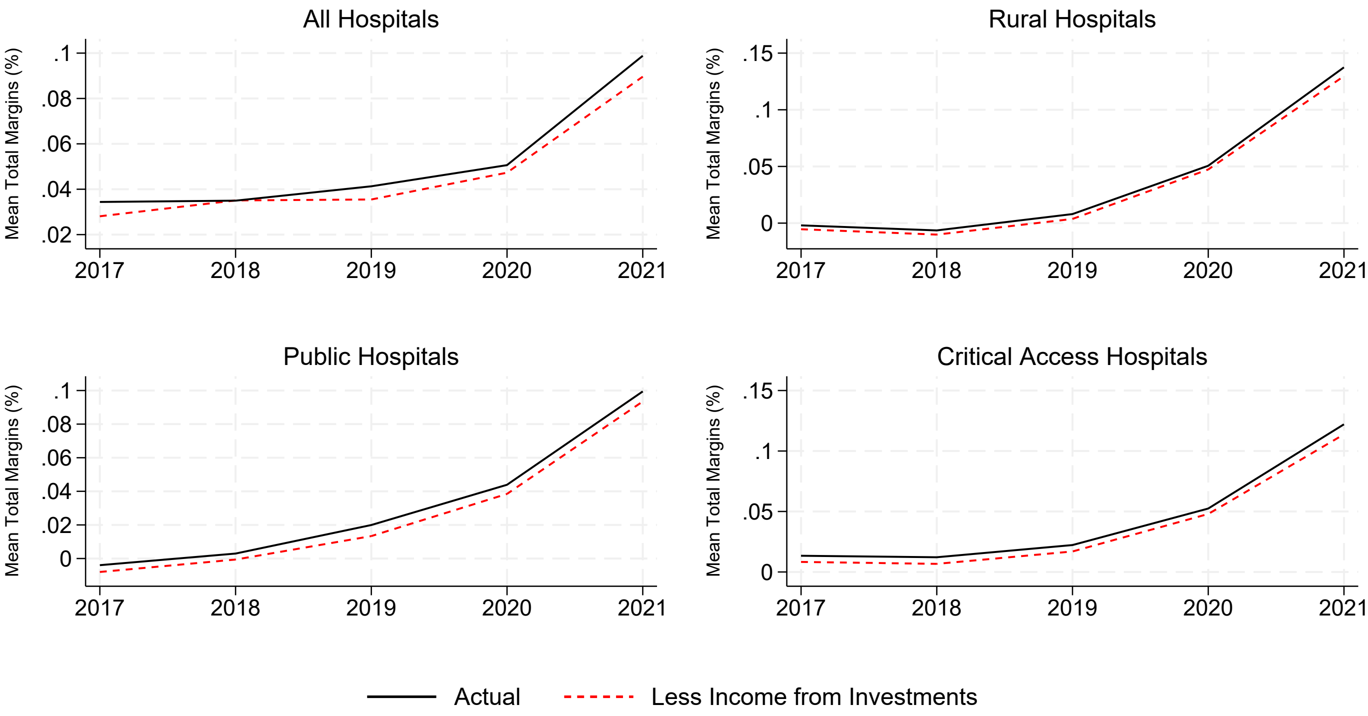

Sources: 2016-2021 HCRIS Cost Report Data; U.S. Department of Agriculture.

EXHIBIT S3: Relationship between Baseline (2019) Total Margins and the Change in Total Margins (2019 to 2021) by Hospital Institutional Characteristics

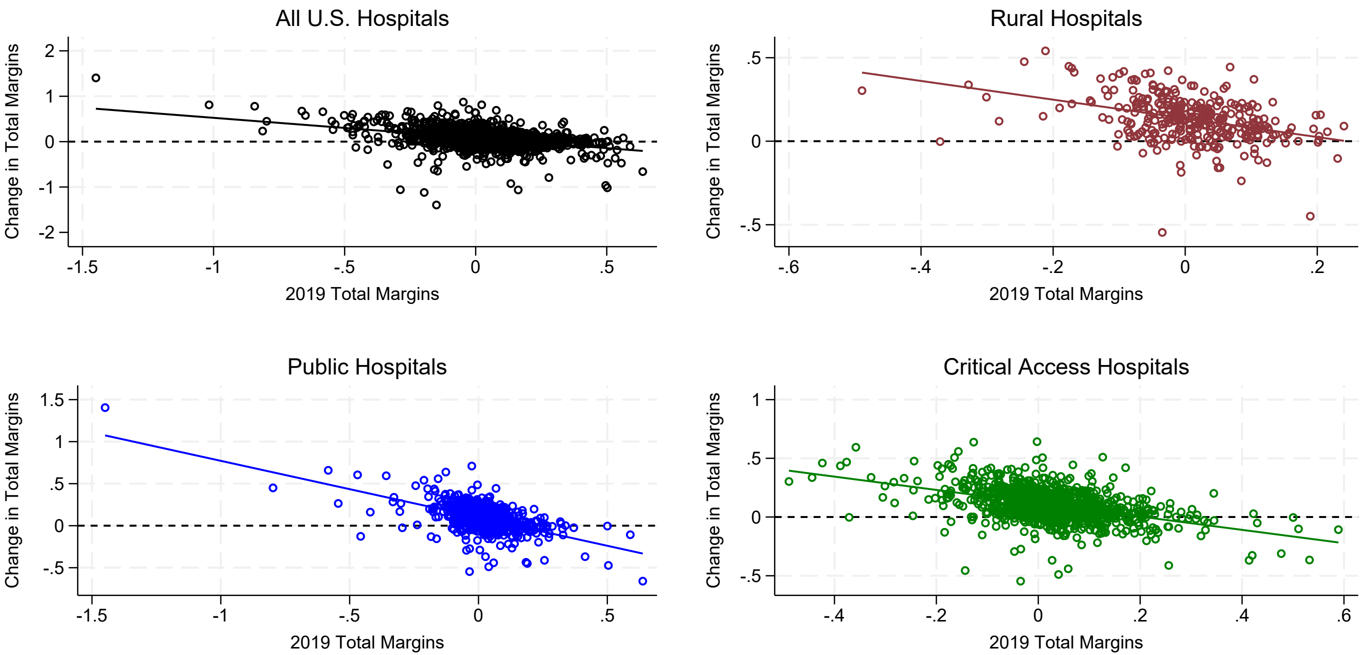

The unit of analysis is the hospital. The vertical axis represents the percentage point change in total margins from 2019 to 2021. Each plot contains a linear best fit line and a horizontal line that distinguishes between an increase (above) or a decrease (below) in total margins over time. The figure is limited to those hospitals that reported total margins in both 2019 and 2021. For the purposes of scaling, the figure excludes four hospitals that experienced > |150%| change in total margins from 2019 to 2021.
Source: 2016-2021 HCRIS Cost Report Data; U.S. Department of Agriculture.

EXHIBIT S4: Statistical Relationship between Baseline (2019) Total Margins and the Change in Total Margins (2019 to 2021)

	Outcome: Change in Total Margins (2019 to 2021)					
	(1)	(2)	(3)	(4)	(5)	(6)
2019 Total Margins	-0.535***	-0.528***	-0.513***	-0.522***	-0.525***	-0.534***
	(0.0625)	(0.0638)	(0.0676)	(0.0674)	(0.0679)	(0.0667)
Hospital Institutional Characteristics						
Rural Hospital			0.0325***	0.0287***	0.0286***	0.0218***
			(0.00735)	(0.00764)	(0.00801)	(0.00658)
Public Hospital			-0.00568	-0.00972	-0.0122	-0.00761
			(0.00763)	(0.00782)	(0.00778)	(0.00820)
Critical Access Hospital			0.0401***	0.0349***	0.0347***	0.0275***
			(0.00858)	(0.00889)	(0.00874)	(0.00856)
Region						
Northeast				-0.0360**	-0.0378**	
				(0.0169)	(0.0182)	
Midwest				0.0227	0.0246	
				(0.0142)	(0.0171)	
South				0.0137	0.0151	
				(0.0145)	(0.0140)	
COVID-19 State Excess Deaths Tertile						
Tertile 2					-0.0135	
					(0.0100)	
Tertile 3					-0.00759	
					(0.0130)	
Hospital Reporting Period Begin Month Fixed Effect		X	X	X	X	X
State Fixed Effects						X
Observations	3,898	3,898	3,898	3,898	3,813	3,898

Standard errors clustered at the state-level.

*** denotes statistical significance at the 1% level; ** denotes statistical significance at the 5% level.

Notes: Sample excludes hospitals with fiscal year reporting periods that differ across data years. COVID-19 state excess deaths tertiles are assigned following the methodology employed by Wang, Bai, and Anderson (2023), which relies on data presented in Woolf et al. (2020). Hospitals located in North Carolina are excluded from this analysis because of delays in reporting.

Source: 2016-2021 HCRIS Cost Report Data; U.S. Department of Agriculture; Woolf et al. (2020).
